# Hydroxyurea-Induced miRNA Expression in Sickle Cell Disease Patients in Africa

**DOI:** 10.3389/fgene.2019.00509

**Published:** 2019-05-28

**Authors:** Khuthala Mnika, Gaston K. Mazandu, Mario Jonas, Gift D. Pule, Emile R. Chimusa, Neil A. Hanchard, Ambroise Wonkam

**Affiliations:** ^1^Division of Human Genetics, Department of Pathology, Faculty of Health Sciences, University of Cape Town, Cape Town, South Africa; ^2^African Institute for Mathematical Sciences, Cape Town, South Africa; ^3^Department of Molecular and Human Genetics, Baylor College of Medicine, Houston, TX, United States

**Keywords:** sickle cell disease, fetal hemoglobin, hydroxyurea, miRNA, Africa

## Abstract

Hydroxyurea (HU) is clinically beneficial in sickle cell disease (SCD) through fetal hemoglobin (HbF) induction; however, the mechanism of HU is not yet fully elucidated. Selected miRNAs have been associated with HU-induced HbF production. We have investigated differential HU-induced global miRNA expression in peripheral blood of adult SCD patients in patients from Congo, living in South Africa. We found 22 of 798 miRNAs evaluated that were differentially expressed under HU treatment, with the majority (13/22) being functionally associated with HbF-regulatory genes, including *BCL11A* (miR-148b-3p, miR-32-5p, miR-340-5p, and miR-29c-3p), *MYB* (miR-105-5p), and *KLF-3* (miR-106b-5), and *SP1 (*miR-29b-3p, miR-625-5p, miR-324-5p, miR-125a-5p, miR-99b-5p, miR-374b-5p, and miR-145-5p). The preliminary study provides potential additional miRNA candidates for therapeutic exploration.

## Introduction

Hydroxyurea (HU), the only food and drug administration (FDA) – approved treatment for sickle cell disease (SCD), is beneficial primarily through its ability to induce fetal hemoglobin (HbF) (Platt et al., 1984; Charache et al., 1992; Zimmerman et al., 2004). Clinical trials have shown hydroxyurea to be efficacious for increasing HbF in children, adolescents, and adults with SCA (Charache et al., 1992; Lee and Ambros, 2001; Thornburg et al., 2009). However, the precise mechanism by which HU can induces HbF in patients with SCA is not fully defined. Three main molecular pathways have been reported in HU-mediated response in increase HbF: (i) Epigenetic modifications, and transcriptional events, (ii) Signaling pathways, and (iii) Post-transcriptional pathways with regulation by Small non-coding RNA oligonucleotides (miRNA) (Pule et al., 2015).

miRNA have emerged as ubiquitous and potent molecular regulators that modulate the expression of many protein-coding genes by inhibiting mRNA translation (Lee and Ambros, 2001; Friedman et al., 2009). Multiple miRNAs have been implicated in the regulation of cell differentiation and maturation during hematopoiesis and erythropoiesis (Havelange and Garzon, 2010; Lawrie, 2010; Zhao et al., 2010). A few studies have demonstrated post-transcriptional regulation of HU-mediated γ-globin expression through miRNA in SCD patients; for example, miR-15a and miR-16-1 have been linked via the transcription factor *MYB*3 to elevated HbF(Zhu et al., 2014; Pule et al., 2016), and expression of miR-26b and miR-151-3p have both been associated with HbF levels at the maximum tolerated dose (MTD) (Walker et al., 2011).

Studies have shown that miRNA expression of erythrocytes contributes to the majority of the miRNA expressions in whole blood (Juzenas et al., 2017). Because recent studies have identified miRNAs in mature erythrocytes that may reflect miRNA regulated processes during early erythropoiesis (Walker et al., 2011), we investigated differential HU-induced miRNA expressions using peripheral blood isolated from SCA patients before starting HU and after reaching the MTD. We identified novel miRNA expression changes after HU treatment, and their associated pathways, which mainly implicate HbF-regulatory genes. Our findings thus, provide novel insights into post-transcriptional mechanisms of actions of HU.

## Materials and Methods

### Ethics Statement

The study was performed in accordance with the Declaration of Helsinki and with the approval of the Faculty of Health Sciences Human Research Ethics Committee, University of Cape Town (HREC Ref. No. 132/2010). Informed and written consent was obtained from all patients that were all adult participants (>18 years).

### Patients and HU Exposure

Ten patients were enrolled in this study, all attending adult hematological clinic of Groote Schuur Hospital in Cape Town (South Africa), denoted as GS01 to GS10. All consenting patients were selected, socio-demographic and clinical data were collected by means of a structured questionnaire. Adult SCA patients were interviewed; patients’ medical records were reviewed, to delineate their clinical features over the past 3 years. Anthropomorphic variables (body mass Index (BMI), and blood pressures (BP) were measured in the outpatient setting. No incentive was provided for participation in the study. Only patients who, who was at steady clinical state, without current acute such as vaso-occlusive painful crisis and had not received a blood transfusion or hospitalization in the past 6 weeks where included. The hematological measures were those reported at the first visit to the hospital (Supplementary Table S1). Two patients GS01 and GS04, were investigated at two stage: before administration and after HU at MTD (indexed as H); Six patients were already on HU at MTD at the time of the study (GS02, GS03, GS07 GS08, and GS09, and GS10); and lastly, two patients (GS05 and GS06) had never been on HU.

### Molecular Method

#### Genotyping: Sickle Cell Disease Mutation, β-Globin Gene Cluster Haplotypes, and 3.7 kb α-Globin Gene Deletion

DNA was extracted from peripheral blood, following instructions on the available commercial kit [QIAamp DNA Blood Maxi Kit. ^®^(Qiagen, United States)]. Molecular analysis to determine the presence of the sickle mutation was carried out by polymerase chain reaction (PCR), followed by DdeI restriction analysis (Saiki et al., 1985). Using published primers and methods, five restriction fragment length polymorphism (RFLP) sites in the β-globin gene cluster were amplified to analyze the *HBB* haplotype background (Bitoungui et al., 2015). The 3.7 kb α-globin gene deletion was screened using expand-long template PCR, as previously reported (Rumaney et al., 2014).

#### RNA Extraction and miRNA Expression Profile

**FIGURE 1:**
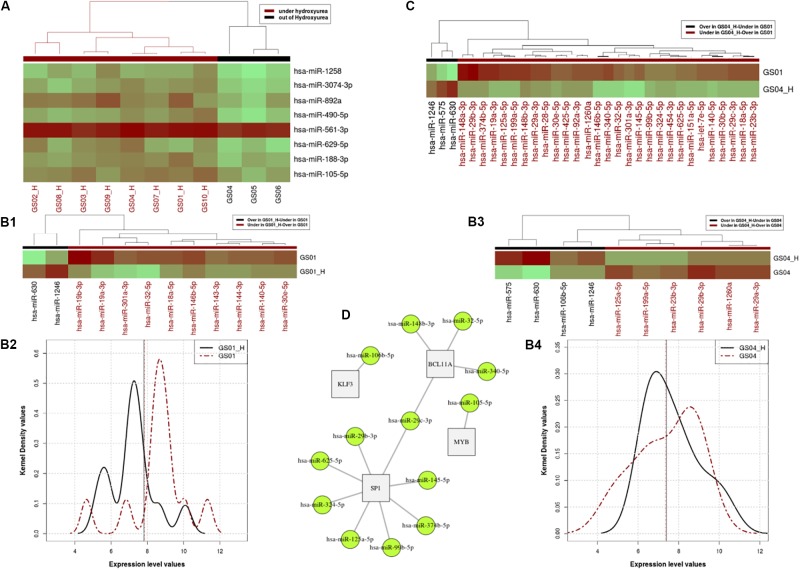
Profiles of Hydroxyurea-induced miRNA expression in selected SCD patients. Panel **(A)**. Heatmap of the miRNA expressing profile for patients that are under HU and off HU in the cross-sectional analysis. Panels **(B,C).** miRNA expressing profile for patients that are under HU and out of HU, excluding the relapsed patient in pair-wire analysis of patients GS01 and GS04. Using the paired Wilcoxon rank test, we found a significant difference between expression profiles of the two states (*p* = 0.032e-2), with distinctive micro-RNAs differentially over or expressed in patients GS01 (Panel **B1**) and GS04 (Panel **B3**), as shown in the heat map (Panel **B2**) and the kernel density distributions (Panel **B4**) plots. Panel **(C).** miRNA expression levels of 29 miRNA that are over or under-expressed in GS04-HU vs. GS01. Panel **(D).** Networks linked to over and under expressed miRNA under HU treatment. In the figure 13 miRNAs target mainly 4 genes *BCL11A*, *MYB*, *KLF3*, and *SP1*, that belong to the same network and influence erythropoiesis and HbF expression.

Total RNA was isolated using the miRNeasy kit according to protocol of the Manufacturer (QIAGEN, Hilden, Germany); and sequenced by the Genomic and RNA Profiling Core at Baylor College of Medicine, United States, using the NanoString Platform (NanoString Technologies, Inc., Seattle, WA, United States), according to manufacturer’s instructions. miRNA expression profile analyses were performed using the significance analysis of microarrays (SAM) tool (Thusher et al., 2001). A cross-sectional analysis was performed for differential expression for all the patients without HU and those under HU at MTD (Table 1 and Figure 1A). In addition, a pair-wise analysis was performed for patients GS01 and GS04, before and after treatment of HU at MTD for each patient alone (Figure 1B1–B4), and for both patients together (Figure 1C), looking mainly for miRNAs that were over or under-expressed, using the paired Wilcoxon rank test. Differences in expression counts of differentially expressed miRNAs were tested using one-factor analysis of variance (ANOVA), after normalizing different samples based on their Fisher-Pearson skewness coefficient scores (Doane and Seward, 2011), adjusted for multiple comparisons with the significance level set to 0.05. We refer the interested readers to the Supplementary File (Section S2, sub-section 2) for more information. Specially, for pair-wise analysis, we extracted sets of over- and under-expressed miRNAs in different sample pairs e.g., GS01-GS01_H, GS01-GS04_H, and GS04-GS01_H using Pearson-Chi square scores and these sets were assessed using sample randomization to check whether the identified sets of over- and under-expressed miRNAs were more than expected by chance (Yocgo et al., 2017).

**Table 1:** Differentially expressed microRNAs between SCD patients on HU and off HU in cross-sectional analysis.

microRNA-ID Fold-change *q*-value (%) *p*-values		
**microRNA**	**microRNA-ID Fold-change**	***q*-values**	***p*-values**

hsa-miR-105-5p	1.566	35.526	0.01644
hsa-miR-188-3p	1.525	35.526	0.01737
hsa-miR-561-3p	1.451	35.526	0.01757
hsa-miR-3074-3p	1.509	35.526	0.01871
hsa-miR-892a	1.327	35.526	0.02010
hsa-miR-490-5p	1.532	35.526	0.02534
hsa-miR-1258	1.339	35.526	0.02519
hsa-miR-629-5p	1.183	35.526	0.02802


*In order to avoid possible residual effect of previous HU exposure Patient GS01 was remove from this analysis for non-compliance at initial administration of HU that he stopped for 3 months, before resuming treatment to achieve MTD.*

### Bioinformatics Pathway Analysis: HU Effects and Identifying Potential Biological Targets

All the differentially expressed miRNAs in cross-sectional analysis (Figure 1A), and miRNAs over- or under-expressed miRNAs in pair-wise analysis (Figure 1B1,B2,C) were used to retrieve potential post-transcriptionally regulated gene targets, from the miRTarBase database (Chou et al., 2015), which stores experimentally validated miRNA-target interactions. For specific miRNAs that were over- and/or under-expressed in different sample pairs, we performed enrichment analyses, using Gene Ontology (GO) process, the protein GO Annotation (GOA) mapping and the Kyoto encyclopeadia of genes and genomes (KEGG) pathway datasets, in order to identify enriched biological processes and pathways in which gene targets are involved (Mazandu and Mulder, 2013).

## Results

### Patients’ Description

Ten patients were investigated, all migrant from Democratic Republic of Congo, with a median age of 25 (95% CI: 23–26). All patients are homozygous for the Sickle cell mutation (HbSS). Patients receiving HU had higher HbF levels than those without HU treatment (13 vs. 4.4%). Most patients had at least a Bantu haplotype, in the beta-globin genes’ cluster; four patients were heterozygous for the 3.7 kb alpha-globin gene deletion; detailed clinical characteristics are shown in Supplementary Table S1.

### Cross-Sectional Analysis of the miRNA Expression Profiling

A total of 829 miRNAs were sequenced, and 798 that passed quality control were analyzed (Supplementary File,Section S2 for more details). The cross-sectional analysis identified 8 miRNAs differentially (over-) expressed with statistical characteristics and expression levels shown in Table 1, and the heat map in Figure 1A, respectively.

### Pair-Wise Analysis of Differential Over-Under-Expressed miRs in Two SCD Patients

With or without HU exposure, we found a significant difference between expression profiles of the two states (*p*-value = 0.03266e-2), with 12 and 10 distinctive micro-RNAs differentially (over or under) in patients GS01 and GS04, respectively (Figure 1B1–B4). In order to elucidate miRNAs that influence the difference between the two patients’ expression level profiles, we investigated miRNA expression levels which are over or under-expressed in GS04_H vs. GS01. A total of 29 miRNAs met these criteria, most were under-expressed in GS04_H (Figure 1C).

### Genes Targets and Biological Pathways of miRNAs That Are Differentially Expressed Under HU Treatment

Next, we used miRNAs that were differentially expressed in cross-sectional analysis of all patients, alongside over- and under-expressed miRNAs identified in pair-wise analysis of GS01 and GS04, to retrieve potential post-transcriptionally regulated genes using datasets extracted from the miRTarBase database (Chou et al., 2015). We found 13 miRNAs that mainly targeted mainly 4 genes *BCL11A*, *MYB*, *KLF3*, and *SP1*, belonging to the same network and predicted to influence erythropoiesis and HbF expression (Figure 1D); most of these miRNAs were under-expressed with the exposure to HU at MTD (Supplementary Table S4).

Additionally, we used genes targeted by miRNAs that were differentially expressed, to identify enriched biological processes and pathways in which targeted genes are involved. We mostly found association with cancer pathways. Other enriched biological pathways identified were *pyrimidine metabolism* (*p*-value = 0.00986), *pathogenic Escherichia coli infection* (*p*-value = 0.00072) and *Oxidative phosphorylation* (*p*-value = 0.00032). Enriched biological process identified with p-adjusted using Bonferroni multiple corrections was miRNA mediated inhibition of translation, the main post-transcriptional mode of action of miRNA (GO: 0035278 with *p*-value adjusted = 0.0274).

## Discussion

The present study is the first to investigate *in vivo* miRNA expression in SCD patients in Africa, exposed to HU. MiRNA expression of erythrocytes is different from that of reticulocytes and leukocytes, but contribute to the majority of the microRNA expression in whole blood (Chen et al., 2008; Juzenas et al., 2017). This supports the most practical approach of using peripheral blood, in this study. Most of the miRNAs found to be differentially expressed under HU treatment in the current study, were also previously shown to be preferentially expressed in erythrocyte in SCD patients (Chen et al., 2008).

A major finding of the present study is the identification of specific and novel miRNA that are targeting HF- regulating genes (Figure 1D), i.e., miR-125b (*SP1*), mi199a, miR-7e, miR-106a, and miR-106b (*KLF3*), miR-140 miR-146; miR-188, miR-143, miR-125a, miR-19b, and miR-105 (*MYB*), miR-23b and miR-29a (*BCL11A* and *SP1*). We replicated previous findings that miR-148a, miR-29a, and mi151-3p, are differentially expressed in CD71+ erythroid cells, both before HU and after HU treatment at MTD in SCD-HbSS patients (Walker et al., 2011). Several other miRNAs are able to increase *γ-*globin gene expression, such as Lin28B, miR-486-3p, with let-seven family participating in the regulation of fetal to adult erythroid development process by increasing *γ-globin* gene expression through inhibitory effects on *BCL11A* (Lee et al., 2013; Ginder, 2015). miR-15a/16-1 restrain the MYB factor which then cause loss of the inhibitory effect on γ-gene and induce HbF in early erythroid progenitors (Sankaran et al., 2011).

Multiple miRNAs that target *SP1* and *KLF3* were differentially expressed under the HU treatment; several of these are novel (Figure 1D) and will require further functional investigation. *KLF3* and *SP1* are transcription factors, that belong to the family of *β-like globin* gene transcription regulation that act by binding to the LCR regions of the 𝜀, γ, and *β-globin* promoters (Hu et al., 2007). *SP1* has been shown to be the main target for miR-23a which increases γ and 𝜀 globin expression by SP1 inhibition and repression. KLF3 factor, a negative regulator of erythropoiesis process, is also specifically inhabited by miR-27a (Ma et al., 2013). Therefore, this translational study provides additional candidates miRNAs that may contribute to globin gene expression and subsequent HbF production, and thus stand as prospects for future post-transcriptional therapeutic approaches that could minimize the alterations of the whole cellular transcriptome and related HU sides effects.

Association of differentially expressed miRNA with cancer pathways might be because cancer pathways are over –represented in the supporting literature. Other enriched biological pathways included “biological process’ associations with Pathogenic *Escherichia coli* infection, Pyrimidine metabolism and Oxidative phosphorylation. These pathways could be related to the known increased susceptibility to bacterial infection in patients with SCD, folate acid metabolism that is important erythropoiesis, or the oxidative stress associated with recurrent vaso-occlusive crisis (VOC), and deserve additional investigations in much larger samples.

There are a few limitations to the present study, the first of which is the modest sample size that might result false positive associations and over-claiming significance. With a larger sample size, it is also possible that additional microRNA and biological pathways would be identified. We have provided a simulation of the needed statistical power in future studies in the section S3 of the Supplementary File provided. Even though the current pilot study did not achieve the expected statistical power for its modest sample size, the results obtained are consistent with the literature, biologically relevant, and provide strong hypothesis for future studies. The second possible limitation is that by analyzing miRNAs that are likely from late-stage erythroblasts instead of erythroid progenitors from the bone marrow, epigenetic or molecular changes resulting from hydroxyurea treatment may have been missed. Lastly, the observed associations with targeted HbF genes regulators in pathway analysis, do not provide direct evidence for miRNA expression with HbF production. Despite these limitations, the significant associations of a limited number of differentially expressed miRNAs that potentially target HbF gene regulators provide preliminary hypothesis-generating results that can be used to design future functional experiments. These results also emphasize the need for future studies to investigate epigenetic processes in mechanisms of HbF expression and induction.

## Conclusion

The study has shown that the global analysis of microRNA expression in peripheral blood of SCD patients, in the African context, can provide valuable insights into the mechanism of action of HU treatment. The study has identified novel HU-induced miRNA that specifically target HbF regulatory genes (*BCL11A*, *MYB*, *KLF-3,* and *SP1*), and are therefore strong candidates for post-transcriptional therapeutic exploration in SCD.

## Ethics Statement

The study was performed in accordance with the Declaration of Helsinki and with the approval of the Faculty of Health Sciences Human Research Ethics Committee, University of Cape Town (HREC Ref. No. 132/2010). Informed and written consent was obtained from all patients that were all adult participants (>18 years).

## Author Contributions

AW conceived and designed the experiments. AW, KM, GP, and NH performed the experiments. AW, GP, and KM patient recruitment, samples and clinical data collection and processing. MJ, GM, EC, KM, NH, and AW analyzed the data. AW, EC, MJ, and GM contributed reagents, materials, and analysis tools. KM, GP, GM, and AW wrote the manuscript. All authors revised and approved the manuscript.

## Conflict of Interest Statement

The authors declare that the research was conducted in the absence of any commercial or financial relationships that could be construed as a potential conflict of interest.
