# Supplementary material for: Hydroxyurea-Induced miRNA Expression in Sickle Cell Disease Patients in Africa
Source: Front Genet. 2019 May 28;10:509. doi: 10.3389/fgene.2019.00509 (PMC6568309; doi:10.3389/fgene.2019.00509)
Supplement: Supplementary file 1 [file Data_Sheet_1.PDF]

## Section S1: General description of patients' information

The clinical characteristics and haematological indices of the miRNA array cohort collected from the Groote Schuur Hospital in Cape Town (South Africa) are shown in **Table S1**.

All consenting patients were selected, socio-demographic and clinical data were collected by means of a structured questionnaire. Adult SCA patients were interviewed; patients' medical records were reviewed, to delineate their clinical features over the past three years. Anthropomorphic variables (body mass Index (BMI), and blood pressures (BP) were measured in the outpatient setting. No incentive was provided for participation in the study. Only patients who, who was at steady clinical state, without current acute such as vaso-occlusive painful crisis and had not received a blood transfusion or hospitalization in the past 6 weeks where included.

Cohort consisted of 10 SCD patients identified GS01 to GS10: with two patients GS01 and GS04 investigated at two stages: before HU and after HU administration at the maximum tolerated dose (MTD) indexed by H; Six patients were already on HU at MTD at the time of the study (GS02, GS03, GS07 GS08 and GS09, and GS10); and lastly, two patients (GS05 and GS06) had never been on HU. Note that for GS01, before HU administration stage corresponds to the non-compliance with the HU treatment for the patient GS01 for about three months

The median age of the patients on HU was 33 years whereas that of patients off HU was 24 years. There was 50 % distribution of females and males on the patients off HU and males were over-represented in the group that was on HU 66.67% were on HU at the time of study enrolment.

The Bootstrap technique [1] was used to compute statistical parameters and p-values between the two independent cohorts of SCD patients on HU and off HU under the null hypothesis that on HU clinical parameter values are greater than those of off HU. Results suggest that some of on HU clinical parameter values, including age, RBC, HB and HbF, were significantly greater than those of off HU (with p-values $> 0.5$). This is not the case for the following clinical parameters: MCV, MCH and PLT, for which data did not show evidence that on HU parameter values are greater than off HU parameter values. Note that there was not enough data to perform similar approach for HbA and HbA2. Finally, a $\chi^2$ independence

test for homogeneity of proportions was performed for Alpha-thalassemia and Haplotype variables, revealing that the two groups are not homogeneous.

**Table S1: Descriptive data for patients who are on HU and off HU.**

| | | No HU (N=4) | Under HU at MDT (N=8) | |
|---|---|---|---|---|
| Variables | | Median (25th-75th percentiles) or % | Median (25th-75th percentiles) or % | P-value |
| Age (Years) | | 24 (23.2-25.5) | 33 (23.2-33.0) | 0.861 |
| Gender (N) | M/F | 2/2 | 1/7 | |
| Haematological Index | RBC (1X1012/ul) | 3.0 (2.7-3.9) | 3.2 (2.3-3.2) | 0.636 |
| | HB (g/dL) | 8.4 (7.9-9.1) | 9.0 (7.4-9.05) | 0.720 |
| | MCV (fL) | 83.8 (65.9-85.9) | 80.9 (74.2-80.9) | 0.374 |
| | MCH (pg) | 29.8 (22.5-30.0) | 28.5 (25.2-28.5) | 0.364 |
| | PLT (1X109/ul) | 368.5 (308.5-622) | 315.5 (188.5-315.5) | 0.073 |
| | HbA (%) | 3.2 | 3 | - |
| | HbA2 (%) | - | 6.6 | - |
| | HbF (%) | 4.4 (4.1-4.7) | 13 (6.9–14·1) | > 0.99 |
| Alpha-thalassemia (%) | αα/αα | 25 (n = 1) | 80 (n = 5) | $2.07 \times 10^{-14}$ |
| | αα/α3.7 | 75 (n = 3) | 20 (n = 1) | |
| Haplotype (%) | Bantu/Benin | 25 | 20 | $5.86 \times 10^{-05}$ |
| | Bantu | 50 | 40 | |
| | Bantu/Atypical | 25 | 20 | |
| | Atypical | | 20 | |

RBC: red blood cell counts; Hb: hemoglobin; MCV: mean corpuscular volume; MCHC: mean corpuscular hemoglobin concentration; WBC: white blood cell counts; PLT: platelet; HbA: adult hemoglobin; HbA2: hemoglobin A2; HbF: fetal hemoglobin;Bantu/Benin; Bantu; Bantu/Atypical.

## Section S2: miRNA expression based on knowledge inference

Different analyses were performed to account for the non-compliance with the HU treatment for the patient GS01 for about three months. Three assumptions were made: (1) the effect of the therapy is not known, in this case, this patient is removed from the dataset to avoid potential uncontrollable biases, (2) the therapy still have an effect and we have assumed that the patient still under HU and (3) the patient is at the state where the effect of therapy has completely waned, in which case, the patient is considered to be out of treatment. Out of 828 miRNAs initially sequenced, only 798 that passed quality control based on the variability of

expression levels tested using Muller statistic [2], testing coefficient of variation ($C_V$) with $C_V < 1$ for low variability and $C_V > 1$ for high variability. These 798 were analysed to identify differentially expressed miRNAs and used to predict post-transcriptionally regulated genes.

## 1. Identifying differentially expressed miRNA

In each of the three assumptions stated above, we performed the expression profile analyses using the SAM tool [3] in order to predict differentially expressed profiles or microRNAs.

For the first assumption, we obtained 8 differentially (over-) expressed microRNAs with statistical characteristics and expression levels are shown in Table 1 and in the heat map in Figure 1A (Main manuscript), respectively.

For (2), results obtained show evidence of 9 differentially (over-) expressed microRNAs, with statistical features in **Table S2** and heat map expression levels in (**Figure S2**)

**Table S2: Differentially expressed microRNAs between SCD patients on HU and off HU including GS01.**

**microRNA-ID Fold-change q-value (%) p-values**

| microRNA | microRNA-ID Fold-change | q-values | p-values |
|---|---|---|---|
| **hsa-miR-561-3p** | 1.457 | 23.183 | 0.01395 |
| **hsa-miR-105-5p** | 1.544 | 23.183 | 0.011616 |
| **hsa-miR-892a** | 1.328 | 23.183 | 0.01641 |
| **hsa-miR-188-3p** | 1.504 | 23.183 | 0.01902 |
| **hsa-miR-1258** | 1.351 | 23.183 | 0.01976 |
| **hsa-miR-1299** | 1.303 | 23.183 | 0.02015 |
| **hsa-miR-3074-3p** | 1.577 | 23.183 | 0.02189 |
| **hsa-miR-490-5p** | 1.603 | 23.183 | 0.02442 |
| **hsa-miR-1275** | 1.510 | 30.911 | 0.03089 |

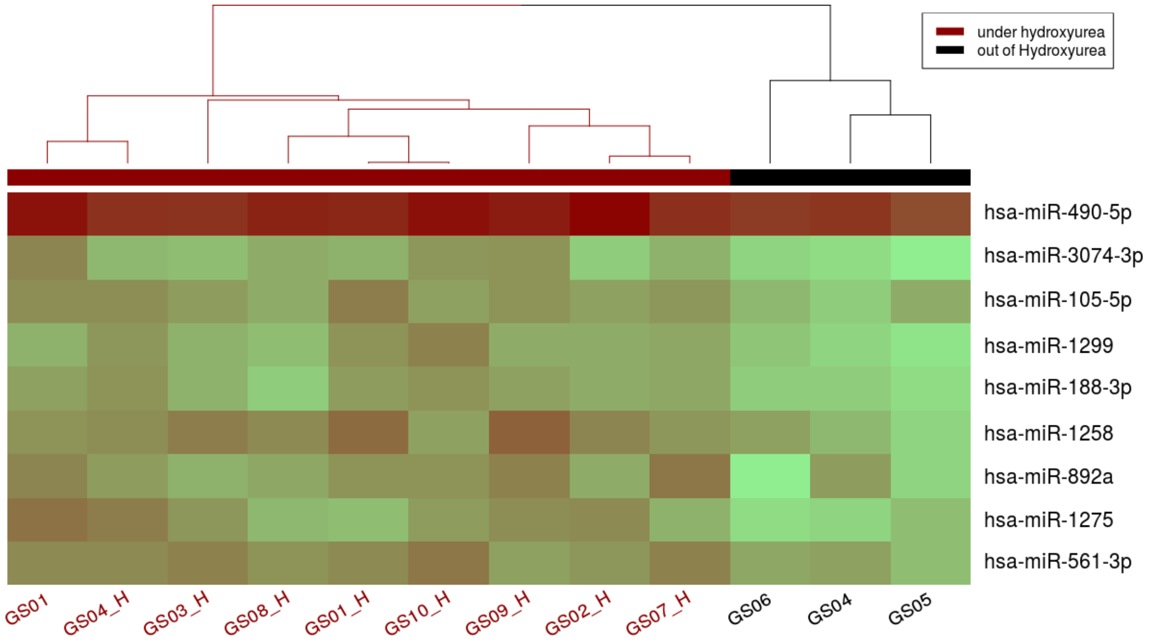


**Figure S1:** miRNA expressing profile for patients that are under HU and out of HU, including the relapsed patient (GS01).

For third assumption, 10 differentially (under-) expressed microRNAs were identified with statistical features in (**Table S3)** and heat map expression levels in (**Figure S3**).

**Table S3: Differentially expressed microRNAs between SCD patients on HU and off HU assuming that GS01 is off HU.**

**microRNA-ID Fold-change q-value (%) p-values**

| microRNA | microRNA-ID Fold-change | q-values | p-values |
|---|---|---|---|
| **hsa-miR-1827** | 0.757 | 54.85 | 0.00364 |
| **hsa-miR-330-5p** | 0.635 | 54.85 | 0.00565 |
| **hsa-miR-1204** | 0.768 | 54.85 | 0.00698 |
| **hsa-miR-422a** | 0.633 | 54.85 | 0.00960 |
| **hsa-miR-579-3p** | 0.719 | 54.85 | 0.01300 |
| **hsa-miR-95-3p** | 0.756 | 54.85 | 0.01524 |
| **hsa-miR-146b-5p** | 0.469 | 54.85 | 0.01762 |
| **hsa-miR-150-5p** | 0.59 | 54.85 | 0.02043 |
| **hsa-miR-613** | 0.795 | 54.85 | 0.02413 |
| **hsa-miR-433-5p** | 0.761 | 54.85 | 0.024403 |

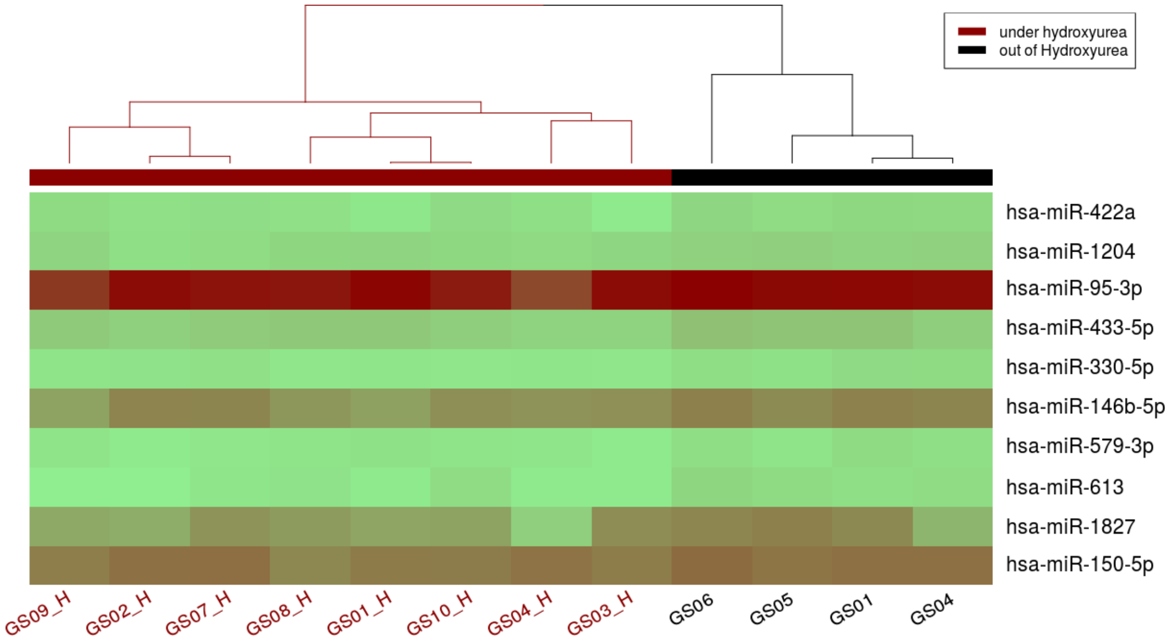


**Figure S3**: miRNA expressing profile for patients that are under HU and out of HU, assuming GS01 is under treatment.

In addition, for the second and third assumptions above, we also focused on the two different states of patient GS01 and GS04, checking whether is difference between the significant differences in expression levels of identified over-expressed or under-expressed miRNAs between the two states using the paired Wilcoxon signed rank test under the null hypothesis that there is no difference between the two state profiles for these two patients. For the context of the second assumption, this mainly aims to check whether results obtained show significant difference in expression levels of identified differentially expressed miRNAs between the two states for GS01. For this patient, the p-value score of 0.7263, which is greater than the significant level set to 0.05, and for GS04, however, the p-value was 0.01427. This suggests that there is no significant difference for GS01, indicating that the previous therapy might still have an effect on the patient.

## 2. miRNA expression based on analysis of HU concentration effect

In order to get more insights on whether GS01 previous therapy still had an influence on the patient and to provide evidence that predicted factors might have contributed to the difference in drug concentration between the two different states of the patient, we explore different datasets based on the differentially expressed miRNAs and results are shown in Figures 3(a) and 3(b), respectively, for GS01 patient after non-complying to the HU therapy and under treatment (GS01_H), the patient GS04 under the treatment (GS04_H) and before the treatment, and two other patients who were still out of treatment (GS05 and GS06).

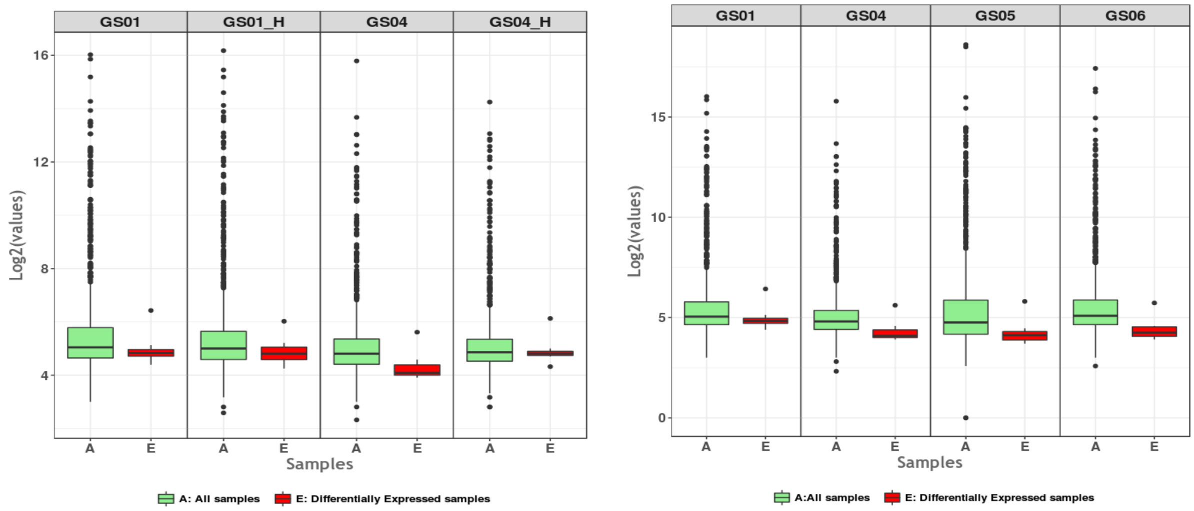


**Figure S4:** Box and whisker plots showing differences in expression level profiles of differentially expressed miRNAs targeting different states of patient GS01 and GS04.

To further confirm this observation, we used graphical representations (boxplots) to explore different datasets based on the differentially expressed miRNAs and results are shown in Figures S3, respectively, for GS01 patient after no-complying to the HU treatment, getting back to treatment (GS01_H) and the patient 4 under treatment (GS04_H), as well as with the patient 4 before the treatment GS04) and two other patients who were still out of treatment (GS05 and GS06). Furthermore, we performed one-way analysis of variance (ANOVA) or one-factor ANOVA to double confirm these results by transforming dataset values in log10 values driven by the Fisher-Pearson skewness coefficient scores [4, 5], which are 1.49025 and 1.29908 in the influence of prior GS01 HU administration or not, respectively, to bring different data subsets into agreement with the normality assumption. These figures and results from the one-way ANOVA still confirmed that there was no significance difference within data subsets (p–value = 0.7723), but there exists a significant difference in miRNA

expression levels between patient GS01, who did not comply to the HU treatment for two months, and those who never got into the HU treatment (p–value = 0.01178). This partly provides evidence that that the effect of HU may still have an influence on a patient relapsing from the treatment for some time.

### 3. Mapping differentially expressed miRNAs to gene targets

Here we used over-under expressed miRNAs identified to retrieve potential post-transcriptionally regulated genes using datasets extracted from the miRTarBase database [6] storing experimentally validated miRNA-target interactions. Different genes targeted and associated miRNAs in miRNA-gene associations shown in Figure 1D in the main manuscript. Table S4 maps differentially expressed miRNAs to gene targets and also provides their expression levels (over or under) based on the exposure to HU at MTD.

**Table S4: Expression profile of miRNAs and their HbF-related target genes**

| microRNA | Target Gene | HU effect on miRs expression level |
|---|---|---|
| **miR-106b-5p** | *KFL3* | Over-expressed |
| **miR-148b-3p** | *BCL11A* | Under-expressed |
| **miR-32-5p** | *BCL11A* | Under-expressed |
| **miR-340-5p** | *BCL11A* | Under-expressed |
| **miR-29c-3p** | *BCL11A* | Under-expressed |
| **miR-29b-3p** | *SP1* | Under-expressed |
| **miR-625-5p** | *SP1* | Under-expressed |
| **miR-324-5p** | *SP1* | Under-expressed |
| **miR-125a-5p** | *SP1* | Under-expressed |
| **miR-99b-5p** | *SP1* | Under-expressed |
| **miR-374b-5p** | *SP1* | Under-expressed |
| **miR-145-5p** | *SP1* | Under-expressed |
| **miR-105-5p** | *MYB* | Over-expressed |

## Section S3: Discussing statistical power of the inferred information

The modest sample size constitutes the main limitation to the present pilot study, which should be addressed in near future as more data are currently being collected. With this larger sample size, it is possible that additional and relevant differentially expressed microRNAs would be identified with a reduced likelihood of selecting false positives. For now, we compute the statistical power score achieved with the current cohort and estimate the sample size needed to achieve the power that may significantly reduce false positive with an optimal effect size.

We computed the statistical power score of two samples with unequal size at the level of significance of 0.05 used throughout our analyses and varying effect size: small (d = 0.2), medium (d = 0.5) and large (d = 0.8) [7]. At these different effect sizes, the power scores achieved were very small with values of 0.05854, 0.10448 and 0.19258 for small. medium and large effect sizes, respectively. The general overview of power score versus sample size is shown in Figure S5, which suggests that, in order to achieve an effective power score of approximately 0.95 for a small effect size (d = 0.2), the size of 650 is needed for each sample type (under HU and off HU administration).

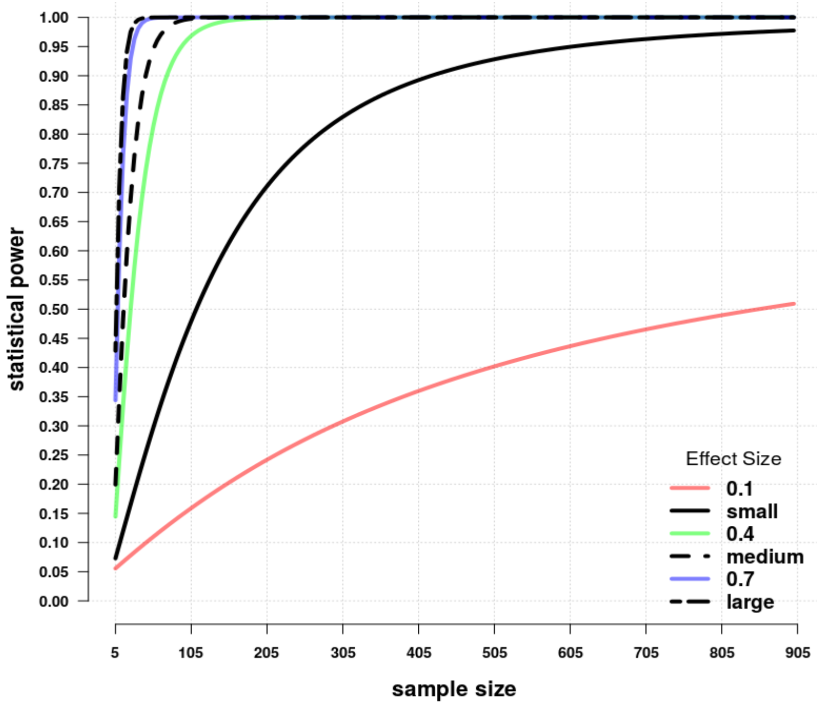


**Figure S5:** Relationships between statistical power and sample size while varying the effect size

It is worth noting that, even though the current pilot study did not achieve the expected statistical power for its modest sample size, the results obtained are consistent with the literature, biologically relevant and promising. These preliminary results are likely to be consistently confirmed on the large data set. As shown in the present study, the differential miRNA expression is largely different for two sample sizes; therefore, we will suggest the use of the median supplement approach in future studies [8].

## References


[1] Li J., Tai B. C., Nott D. J. (2009). Confidence interval for the bootstrap P-value and sample size calculation of the bootstrap test, Journal of Nonparametric Statistics, 21(5):649–661

[2] Miller, G. E. (1991). Asymptotic test statistics for coefficients of variation. Communications in Statistics-Theory and Methods, 20:3351–3363.

[3] Thusher, V. G., and Tibshirani, R. (2001) Significance analysis of microarrays applied to ionising radiation response. Proc Natl Acad Sci U S A. 98, 5116–5221. doi:10.1073/pnas.091062498.

[4] Howell D. C. Statistical Methods for Psychology, 6th edition. Belmont:Thomson Wadsworth; 2007, pp. 318–324.

[5] Tabachnick B. G, Fidell L. S. (2007) Using Multivariate Statistics, 5th edition. Boston: Allyn and Bacon; 2007, pp. 86–89.

[6] Chou, C., Chang, N., Shrestha, S., Hsu, S., Lin, Y., Lee, W., Yang, C., Hong, H. et al. (2015). miRTarBase 2016: updates to the experimentally validated miRNA-target interactions database. Nucleic Acids Res. 44, D239–D247. doi: 10.1093/nar/gkv1258.

[7] Cohen J. (1988). Statistical Power Analysis for the Behavioral Sciences. New York, NY: Routledge Academic.

[8] Adabor E. S., Acquaah-Mensah, G. K. (2017). Machine learning approaches to decipher hormone and HER2 receptor status phenotypes in breast cancer, Briefings in Bioinformatics, bbx138, https://doi.org/10.1093/bib/bbx138
